# Tongue pressure and self-assessment of swallowing after total laryngectomy

**DOI:** 10.1590/2317-1782/e20240185en

**Published:** 2025-03-31

**Authors:** Natália Carminati, Gracielle dos Santos David, Mariana Pinheiro Brendim

**Affiliations:** 1 Departamento de Fonoaudiologia, Universidade Federal do Rio de Janeiro – UFRJ - Rio de Janeiro (RJ), Brasil.

**Keywords:** Laryngectomy, Swallowing, Swallowing Disorders, Tongue, Head and Neck Câncer, Dysphagia

## Abstract

**Purpose:**

To evaluate tongue pressure, self-perception of swallowing, and whether tongue pressure is correlated with self-perception of swallowing in individuals undergoing total laryngectomy.

**Methods:**

Cross-sectional study with two groups – with and without total laryngectomy, matched by age and sex to individuals with total laryngectomy. Participants had their tongue tip and dorsum pressure measured and self-assessed their swallowing with the Swallow Outcomes After Laryngectomy questionnaire (SOAL).

**Results:**

The sample totaled 26 participants, 13 from each group. The mean maximum tongue dorsum pressure was 41.2±18.7 and 27.9±9.3 kilopascals, respectively, in the groups with and without total laryngectomy (p = 0.03). The median maximum tongue tip pressure was 33.7 (23.8-49.3) and 29.1 (22.5-35.7) kilopascals, respectively, in the groups with and without total laryngectomy (p = 0.29). The median SOAL was 6 (2.5-8.5) points in the group with total laryngectomy. The SOAL score was not statistically significantly correlated with tongue tip pressure (r = -0.17; p = 0.58) or dorsum pressure (r = -0.30; p = 0.31).

**Conclusion:**

Individuals with total laryngectomy had higher tongue dorsum pressure, although there was no difference in tongue tip pressure between individuals with and without total laryngectomy. Tongue pressure was not correlated with self-assessment of swallowing, although tongue tip pressure was correlated with dorsum pressure in individuals with total laryngectomy.

## INTRODUCTION

Individuals with advanced laryngeal or hypopharyngeal cancer may require surgical treatment, such as total laryngectomy (TL), with or without radiotherapy or radiochemotherapy^(1)^. TL is the resection of the entire laryngeal framework and requires the definitive separation of the digestive tract from the respiratory tract, resulting in the loss of laryngeal voice and possible changes in swallowing^(1,2)^.

Dysphagia in individuals undergoing TL has a highly variable prevalence and may require significant changes in diet and lifestyle^(3)^. Considering the importance of monitoring signs and symptoms of dysphagia for clinical practice, several studies have investigated the self-assessment of swallowing in these individuals^(2-4)^. Studies in Brazilians have mainly used instruments to assess quality of life^(5-7)^ that were not developed specifically for TL patients. Therefore, they do not always highlight relevant aspects related to swallowing in these individuals^(4)^. The Swallow Outcomes After Laryngectomy questionnaire (SOAL) has been recently translated and adapted to Brazilian Portuguese, allowing the identification and monitoring of swallowing disorder symptoms specifically in the population undergoing TL^(8)^.

The standard TL procedure includes resection of the entire larynx, infrahyoid muscles, and hyoid bone^(2)^. Since several tongue muscles are connected to the hyoid bone^(9)^, it can be assumed that these individuals’ tongue pressure will change. In addition, TL causes anatomical and physiological changes in the pharyngeal structure and movement^(10)^, which can lead to increased resistance of the neopharynx to the flow of the bolus. During swallowing, the bolus is transferred from an area of ​​high pressure to an area of ​​low pressure; hence, the possibility of modifying tongue pressure in these individuals can be reconsidered to overcome the resistance of the neopharynx. Some researchers support this hypothesis, revealing a difference in tongue base pressure in individuals undergoing TL^(11)^.

The literature highlights that these individuals have compensatory tongue movements^(10)^, reduced posterior tongue base movement, and a compensatory increase in pressure in this region to propel the bolus through the neopharynx^(12)^. However, it is not clear whether there is a difference in tongue tip and dorsum pressure between individuals with and without TL. Furthermore, few studies have investigated tongue pressure in individuals undergoing TL. Therefore, this study aimed to assess tongue tip and dorsum pressure, describe self-assessment of swallowing with a specific instrument for TL patients, and verify whether tongue pressure is correlated with self-assessment of swallowing in individuals undergoing TL.

## METHODS

This study was approved by the Research Ethics Committee of the Clementino Fraga Filho University Hospital (HUCFF) under evaluation report number 5.603.176. Participants agreed to participate in the study and signed an informed consent form.

This cross-sectional observational study was carried out at HUCFF’s speech-language-hearing (SLH) outpatient clinic between April 2023 and January 2024.

The inclusion criteria for the study group were adults undergoing TL and followed up at HUCFF’s SLH outpatient clinic. The exclusion criteria for the study group were individuals with neurological disease, cognitive or behavioral changes that prevented them from performing the study procedures, craniofacial malformation, or other head and neck surgery.

The inclusion criteria for the comparison group were adults not undergoing TL, matched by sex and age to the study group. Individuals undergoing treatment at the voice outpatient clinic and family members of patients undergoing SLH therapy at the same hospital were invited to join the comparison group. The exclusion criteria for the comparison group were individuals at risk for dysphagia (e.g., neurological diseases, craniofacial malformation, and head and neck surgery or radiotherapy), risk of dysphagia (cutoff score > 3) identified by the Oropharyngeal Dysphagia Screening in Older Adults (RaDI, in Portuguese)^(13)^, and cognitive or behavioral changes that would prevent them from performing the study procedures. The TL patients at the study institution are older adults. Hence the study used the RaDI, which is appropriate to assess the risk of dysphagia in the comparison group, matched to the study group and likewise composed of older people.

An SLH pathologist investigated the groups’ inclusion and exclusion criteria through electronic medical records and interviewed participants regarding their health conditions.

An SLH pathologist performed the study procedures, measuring the participants’ tongue tip and dorsum pressure, collecting physical and anthropometric data (sex, age, weight, height, and body mass index), and FOIS level^(14)^. They applied the SOAL^(8)^ and collected clinical information (disease staging, treatment modalities, and time since surgery) from the study group participants’ medical records, and applied the RaDI^(15)^ to the comparison group.

Tongue pressure was measured with a lip and tongue pressure biofeedback device (PLL Pro-Fono), extracting the mean pressure (in kilopascals – kPa) in the maximum tongue pressure task. The measurement was taken on two parts of the tongue – first on the anterior part, and then on the tongue dorsum. To measure tongue tip pressure, participants were instructed to hold the bulb with one hand, insert it completely into the oral cavity, and position it on the tip of the tongue. Then, they were instructed to press the air bulb against the palate (alveolar region) with the tip of the tongue with as much force as possible for 3 seconds. To measure tongue dorsum pressure, they were instructed to hold the bulb with one hand, insert it completely into the oral cavity, and position it on the medial tongue dorsum. They were then instructed to press the air bulb against the hard palate with the back of the tongue with as much force as possible for 3 seconds. Three tongue pressure measurements were taken with a 30-second interval between measurements. The average of the three measurements of the tongue tip and dorsum recorded by the equipment was considered for data analysis, in accordance with the method used by other researchers^(16)^.

The sample size was calculated through a pilot study with part of the population of interest, considering an α = 0.05 and a test power (1-β) = 0.80, estimated by the tongue dorsum pressure measurements. The number required was 28 individuals, with 14 in each group.

Data were analyzed using the SPSS program. Categorical data were presented as absolute and relative frequency, while numerical data were presented as mean and standard deviation, in the case of data with normal distribution, or as median and interquartile ranges, in the case of data without normal distribution. Data normality was verified using the histogram and the Shapiro-Wilk test.

Pearson's chi-square test or Fisher's exact test compared categorical data between groups in cases of cells with a frequency lower than five. The independent samples t-test compared numerical data between groups in cases of parametric test indication, and the Mann-Whitney test, in cases of nonparametric test indication. The parametric test was indicated because the premise of normal data distribution was satisfied in both groups. The homogeneity of variance was verified using the Levene test. The Spearman correlation test was used for correlation analysis, due to the indication of a nonparametric test. The level of statistical significance was set at 5% (p < 0.05).

## RESULTS

Two of the 15 TL patients included in the study were excluded, one due to glossectomy and the other due to a diagnosis of dementia. The final sample comprised 13 participants in the study group and 13 in the comparison group. The total RaDI score in the comparison group ranged from 0 to 2 points, with a median of 0 (0-1.5) points. All participants in the study group were TL, and none underwent pharyngolaryngectomy.

The participants’ characteristics are shown in Table 1. There was no statistically significant difference in their characteristics or in the FOIS level between the groups. Regarding the disease staging in the study group, the tumor extension ranged from T2 to T4. The metastasis in regional lymph nodes ranged from N0 and N3. No participant had distant metastasis.

**Table 1:** Characteristics of study group and comparison group participants

Characteristics	Study group	Comparison group	Total	p
	(n = 13)	(n = 13)	(n = 26)	
Male	12 (50%)	12 (50%)	24 (100%)	0.760^a^
Age (years)	67.4 ± 6.9	66.7 ± 7.1	67.0 ± 6.9	0.804^b^
Weight (kg)	69.0 ± 12.3	80.4 ± 16.4	74.7 ± 15.3	0.056^b^
Height (m)	1.72 ± 0.12	1.70 ± 0.08	1.71 ± 0.10	0.617^b^
BMI (Kg/m^2^)	23.9 (20 – 26.2)	26.2 (23.5 – 30.2)	25.6 (21.5 – 27.6)	0.050^c^
FOIS level	7 (6 – 7)	7 (7 – 7)	7 (7 – 7)	0.101^c^
Neck dissection	13 (100%)	-	-	-
Radiotherapy	11 (84.6%)	-	-	-
Chemotherapy	2 (15.4%)	-	-	-
Time since surgery (months)	17 (6.5 – 28.5)	-	-	-
Alaryngeal voice				
- esophageal voice	9 (69.2%)	-	-	-
- electronic larynx	2 (15.4%)	-	-	-
- tracheoesophageal voice	2 (15.4%)	-	-	-

Values are presented as relative and absolute frequencies, mean ± standard deviation, or medians (interquartile range)

a Fisher´s exact test;

b Independent samples t-test;

c Mann-Whitney test

**Caption:** BMI = body mass index

**Figure 1:**
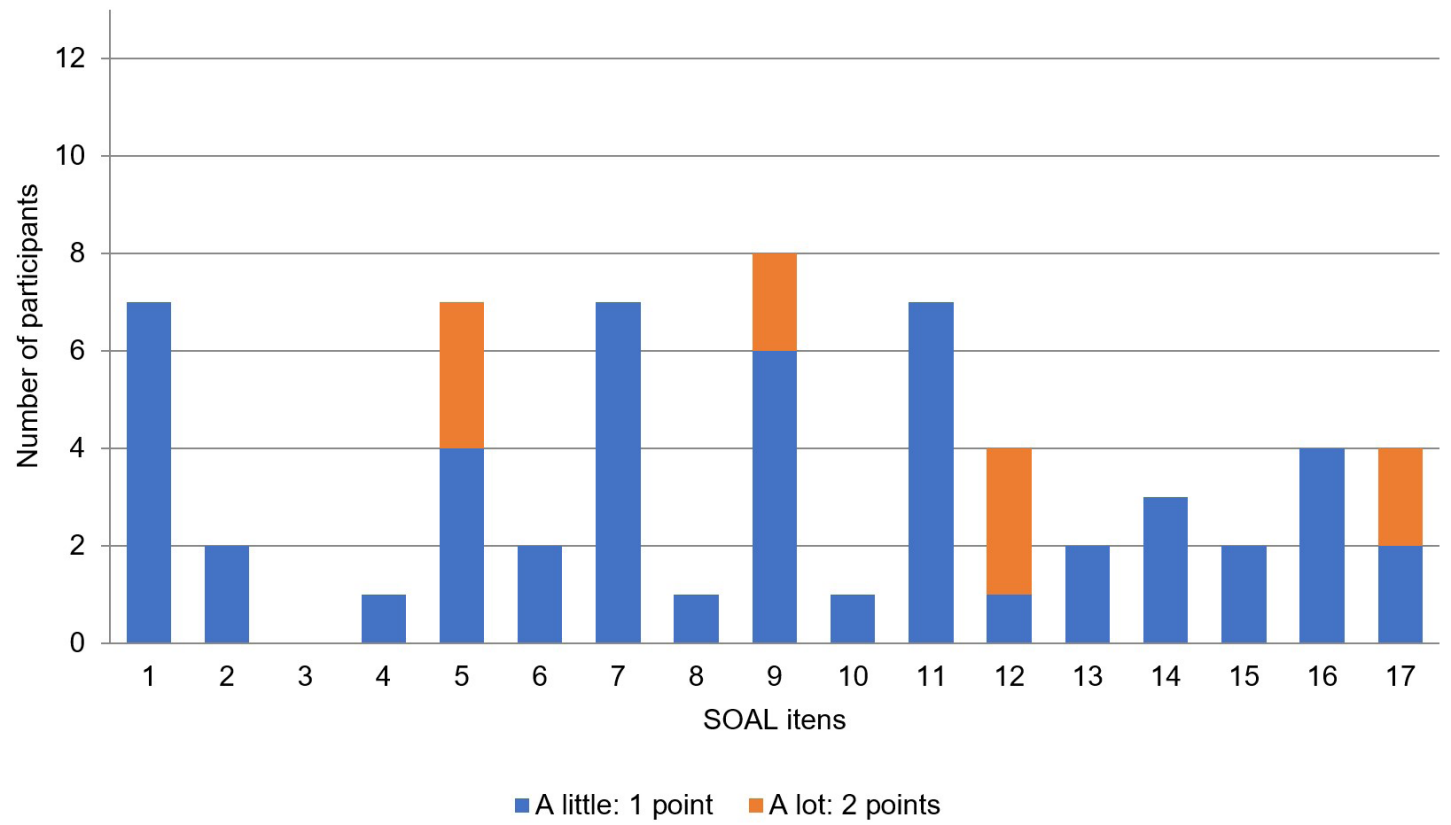
Frequency and intensity of swallowing disorder symptoms

The total SOAL score in the study group ranged from 0 to 10 points, with a median of 6 (2.5-8.5) points. The frequency and intensity of swallowing disorder symptoms, reported by participants through the SOAL, are shown in Figure 1. The most frequent symptoms were related to items 9 (“Do you need to drink liquid to help the food go down?”), present in eight participants, and 1 (“In your opinion, do you currently have a problem swallowing?”), 5 (“Do you have trouble swallowing hard/dry foods [bread rolls, cookies?]”), 7 (“After you swallow, do you feel like food gets stuck in your throat?”), and 11 (“Do you avoid certain foods because you cannot swallow them?”), present in seven participants. The most intense swallowing disorder symptoms reported were related to items 5 (“Do you have trouble swallowing hard/dry foods (bread roll, cookies?”) and 12 (“Do you take a long time to eat a meal?”), reported by three participants, followed by items 9 (“Do you need to drink liquid to help the food go down?”) and 17 (“Do you feel embarrassed eating with other people?”), pointed out by two participants. SOAL item 3 (“Do you have trouble swallowing thickened liquids [creamy soup, smoothies]?”) was the only one not scored (0 points) by any participant.

The groups’ tongue tip and dorsum pressure measurements are presented in Table 2. There was a statistically significant difference in tongue dorsum pressure between the groups, although there was no difference in tongue tip pressure between them.

**Table 2:** Comparison of tongue pressure between groups

Pressure (kPa)	Study group	Comparison group	Total	p
Tongue dorsum	41.2 ± 18.7	27.9 ± 9.3	34.6 ± 16.0	**0.034** a
Tongue tip	33.7 (23.8 – 49.3)	29.1 (22.5 – 35.7)	30.0 (23.2 – 40.8)	0.287^b^

Values are presented as mean ± standard deviation or medians (interquartile range)

a Independent samples t-test;

b Mann-Whitney test

The correlation assessment between tongue pressures and the self-assessment of swallowing in the study group is presented in Table 3. There was no statistically significant correlation between tongue pressure measurements and the self-assessment of swallowing, although there was a moderately strong positive correlation between the tongue tip pressure and the tongue dorsum pressure.

**Table 3:** Correlation between tongue pressure and self-assessment of swallowing

Tongue pressure	Dorsum pressure	SOAL score
Tip pressure	r = 0.665; **p = 0.013**	r = - 0.168; p= 0.583
Dorsum pressure	-	r = - 0.303; p = 0.314

Spearman correlation test

## DISCUSSION

This study measured and compared tongue tip and dorsum pressure in individuals with and without TL. It also described the self-assessment of swallowing of individuals undergoing TL and analyzed whether their tongue pressure measurements were correlated with the self-assessment of swallowing.

The results reveal that individuals submitted to TL had greater tongue dorsum pressure than those without TL. On the other hand, as other researchers, this study did not find a statistically significant difference in tongue tip pressure between individuals with and without TL^(17)^.

Other studies measured the maximum tongue tip pressure in individuals with TL, corresponding to 44.1±11.3 kPa^(9)^ and 50.6 (95% CI 45.1-56.1) kPa^(2)^ – measurements higher than those found in this study. However, these studies used the IOPI, which determines the maximum pressure (peak pressure) achieved, unlike the PLL instrument used in this study, which determines the average tongue pressure during the maximum pressure task.

The tongue is a fundamental structure for swallowing. Besides its important role in the oral phase, its action is decisive for performing the pharyngeal phase. The approximation of the base of the tongue to the posterior pharyngeal wall is crucial for generating pharyngeal pressure and efficient direction of the bolus^(18)^. However, individuals undergoing TL may present impairment of the posterior movement of the tongue base^(12)^. Therefore, the greater tongue dorsum pressure found in this study in TL patients may be a compensatory adjustment developed because of the anatomical and physiological changes in the swallowing function caused by the surgery.

Furthermore, one can consider the importance of tongue dorsum pressure for introducing air into the esophagus to acquire esophageal voice, the most used method in this study population. Thus, the increase in tongue dorsum pressure in these individuals may also be an adjustment for learning esophageal voice. A study found no difference in tongue strength between TL patients proficient in esophageal voice and individuals without TL^(17)^. However, it was found that TL patients who use the electronic larynx may have less tongue strength than individuals without TL^(19)^.

Our results revealed a moderate positive correlation between tongue tip pressure and tongue dorsum pressure in TL individuals – i.e., the higher the tongue tip pressure, the higher the tongue dorsum pressure. This result is easily explained by the fact that these anatomical portions are structurally dependent since they belong to the same anatomical structure. Therefore, individuals with greater pressure in one portion of the tongue are expected to have greater pressure in another portion of this same structure.

The SOAL score ranges from 0 to 34 points, and higher scores indicate greater self-reported problems related to swallowing^(4)^. One study found a mean score of 8.6 in individuals with a normal diet and 18.3 in individuals with a modified diet or without an oral diet^(4)^. According to Govender et al.^(20)^, an individual without adverse characteristics on videofluoroscopy would have a predicted score of approximately 5 points on the SOAL. The median SOAL found in the participants of our study was slightly higher (6 points), which indicates that a considerable portion of these individuals have symptoms related to difficulty in swallowing, but they may not have adverse characteristics on videofluoroscopy. Furthermore, our results revealed a lower SOAL score than that found in other studies, with a mean of 11.3±7.6^(4)^ and 13.6 (95% CI 10.8-16.3) points^(2)^, indicating a worse result in the self-assessment of swallowing in these other studies. One reason that may justify this difference is the time of treatment for the disease, as the late effects of radiotherapy, such as fibrosis and stenosis, may negatively impact swallowing function. While our participants had a median of 17 months after surgery, the other studies had a median of 39^(4)^ and 47 months^(2)^. Another possible justification for this difference is the fact that the participants in our study underwent speech therapy, which may help reduce symptoms of swallowing disorders.

In agreement with the literature, most participants in this study reported symptoms related to difficulty in swallowing, mainly associated with the need to drink liquids to help the food go down^(3)^. Moreover, difficulty swallowing hard or dry foods, food stuck in the throat, and avoiding certain foods because they cannot swallow them were also very common symptoms in our population. These symptoms corroborate the findings of other researchers^(2-4)^ and were expected since the anatomical and physiological changes after TL predispose them to present residue in the neopharynx^(21)^. According to the literature, the increase in the size and viscosity of the bolus implies an increase in pharyngeal residue in TL patients^(22)^. On the other hand, symptoms related to difficulty swallowing liquids, thick liquids, and pureed or soft foods were absent or less frequent in our population, in agreement with other studies^(2,4)^.

Despite our hypothesis that individuals with lower tongue pressure could have worse self-assessed swallowing scores, this study found no correlation between the measurement of tongue pressure and the self-assessed swallowing score in TL individuals. Corroborating these data, other researchers found no difference in peak tongue base pressure between TL individuals with and without symptoms of dysphagia^(22)^.

This study has limitations due to the small sample size. Therefore, there may have been a type II error in the analysis of the comparison of tongue tip pressure between individuals with and without TL and the correlation between tongue pressure and self-assessment of swallowing in TL individuals. Furthermore, this study did not consider the surgical closure technique used in the participants, which may interfere with the biomechanics of swallowing^(22)^ and, consequently, in the aspects evaluated in this study.

## CONCLUSION

The pressure measurements of the tongue dorsum and tip in TL study participants reached, on average, 41 kPa and 34 kPa, respectively. It can be concluded that these individuals have higher tongue dorsum pressure than individuals without TL, although there was no difference in tongue tip pressure between individuals with and without TL participating in this study. Furthermore, tongue pressure was not correlated with self-assessment of swallowing in TL individuals in this study, although tongue tip pressure was correlated with tongue dorsum pressure in them. Most of these individuals had symptoms of swallowing disorders related to the need to ingest liquids to help transport the bolus, difficulty swallowing hard or dry foods, the sensation of food stuck in the throat, and the need to avoid some foods due to swallowing difficulties.
