# Supplementary material for: Tongue pressure and self-assessment of swallowing after total laryngectomy
Source: Codas. 2025 Mar 31;37(3):e20240185. doi: 10.1590/2317-1782/e20240185en (PMC11963879; doi:10.1590/2317-1782/e20240185en)
Supplement: Translation

# Translation: Pressão de língua e autoavaliação da deglutição após laringectomia total

**DOI:** 10.1590/2317-1782/e20240185pt

**Authors:** Natália Carminati, Gracielle dos Santos David, Mariana Pinheiro Brendim

**Affiliations:** 1 Departamento de Fonoaudiologia, Universidade Federal do Rio de Janeiro – UFRJ - Rio de Janeiro (RJ), Brasil.

**Keywords:** Laringectomia, Deglutição, Transtornos de Deglutição, Língua, Câncer de Cabeça e Pescoço, Disfagia

## Abstract

**Objetivo:**

Avaliar a pressão de língua, a autopercepção da deglutição e se existe correlação entre a pressão de língua e a autopercepção da deglutição em indivíduos submetidos à laringectomia total.

**Método:**

Estudo transversal, constituído por dois grupos: com laringectomia total e sem laringectomia total (pareado por idade e sexo aos indivíduos com laringectomia total). Os participantes foram submetidos à mensuração da pressão de ponta e de dorso de língua; e à autoavaliação da deglutição, por meio do questionário *Swallow Outcomes After Laryngectomy* (SOAL).

**Resultados:**

A amostra totalizou 26 participantes, sendo 13 de cada grupo. As médias da pressão máxima de dorso de língua foram 41,2±18,7 e 27,9±9,3 kilopascal, respectivamente, nos grupos com e sem laringectomia total (p=0,03). As medianas da pressão máxima de ponta de língua foram 33,7 (23,8-49,3) e 29,1 (22,5-35,7) kilopascal, respectivamente, nos grupos com e sem laringectomia total (p=0,29). A mediana do SOAL foi 6 (2,5-8,5) pontos no grupo com laringectomia total. Não houve correlação estatisticamente significativa entre a pontuação do SOAL e a pressão de ponta (r=-0,17; p=0,58) e de dorso (r=-0,30; p=0,31) de língua.

**Conclusão:**

Indivíduos com laringectomia total apresentam maior pressão de dorso de língua, apesar de não haver diferença na pressão de ponta de língua entre indivíduos com e sem laringectomia total. Não há correlação entre a pressão de língua e a autoavaliação da deglutição, embora haja correlação entre a pressão de ponta e de dorso de língua de indivíduos com laringectomia total.

## INTRODUÇÃO

Indivíduos com câncer avançado de laringe ou de hipofaringe podem necessitar de tratamento cirúrgico, como a laringectomia total (LT), associada ou não à radioterapia ou radioquimioterapia^(1)^. A LT consiste na ressecção de todo arcabouço laríngeo e requer a separação definitiva entre a via digestiva e a via respiratória, o que resulta na perda da voz laríngea e na possibilidade de alteração da deglutição^(1,2)^.

A disfagia em indivíduos submetidos à LT apresenta prevalência bastante variável e pode acarretar a necessidade de mudanças significativas na dieta e no estilo de vida^(3)^. Considerando a importância do monitoramento dos sinais e sintomas da disfagia para a prática clínica, diversos estudos investigaram a autoavaliação da deglutição desses indivíduos^(2-4)^. Na população brasileira, foram empregados principalmente instrumentos de avaliação da qualidade de vida^(5-7)^, que não foram desenvolvidos especificamente para laringectomizados totais e, portanto, nem sempre destacam aspectos relevantes relacionados à deglutição destes indivíduos^(4)^. Recentemente, foi traduzido e adaptado para o português brasileiro, o questionário *Swallow Outcomes After Laryngectomy Questionnaire* (SOAL), que permite identificar e monitorar os sintomas de transtornos de deglutição especificamente na população submetida à LT^(8)^.

O procedimento padrão de LT inclui a ressecção de toda laringe, músculos infra-hioideos e osso hioide^(2)^. Ponderando que diversos músculos da língua estão ligados ao osso hioide^(9)^, pode-se supor que haja mudança na pressão de língua destes indivíduos. Além disso, a LT acarreta alterações anatômicas e fisiológicas na estrutura e no movimento da faringe^(10)^, que pode levar ao aumento da resistência da neofaringe ao fluxo do bolo alimentar. Sabendo que, durante a deglutição, o bolo alimentar é transferido de uma área de alta pressão para uma área de baixa pressão, pode-se cogitar mais uma vez a possibilidade de modificação da pressão de língua nestes indivíduos para vencer a resistência da neofaringe. Tal hipótese é sustentada por alguns pesquisadores, que revelaram diferença na pressão de base de língua de indivíduos submetidos à LT^(11)^.

Apesar da literatura destacar que estes indivíduos apresentam movimentos compensatórios de língua^(10)^ e indícios de redução do movimento de posteriorização de base de língua e aumento compensatório de pressão nessa região para impulsionar o bolo pela neofaringe^(12)^, não está claro se há diferença na pressão de ponta e de dorso de língua entre indivíduos com LT e sem LT. Ademais, poucos estudos investigaram a pressão de língua em indivíduos submetidos à LT. Sendo assim, o objetivo deste estudo foi avaliar a pressão de ponta e de dorso de língua, descrever a autoavaliação da deglutição, por meio de um instrumento específico para laringectomizados totais, e verificar se existe correlação entre a pressão de língua e a autoavaliação da deglutição em indivíduos submetidos à LT.

## MÉTODO

Estudo aprovado pelo Comitê de Ética em Pesquisa do Hospital Universitário Clementino Fraga Filho (HUCFF): parecer número 5.603.176. Os participantes concordaram em participar do estudo e assinaram o Termo de Consentimento Livre e Esclarecido.

Trata-se de um estudo observacional transversal, realizado no ambulatório de Fonoaudiologia do HUCFF, no período entre abril de 2023 e janeiro de 2024.

Os critérios de inclusão para o grupo de estudo foram indivíduos adultos, submetidos à cirurgia de LT e acompanhados no ambulatório de Fonoaudiologia do HUCFF. Os critérios de exclusão para o grupo de estudo foram indivíduos com doença neurológica, alteração cognitiva ou comportamental que impedisse a realização dos procedimentos do estudo, malformação craniofacial ou outra cirurgia em região de cabeça e pescoço.

Os critérios de inclusão para o grupo de comparação foram indivíduos adultos, que não tenham sido submetidos à cirurgia de LT e pareados por sexo e idade ao grupo de estudo. Foram convidados para compor o grupo de comparação, indivíduos em atendimento no ambulatório de Voz e familiares de pacientes em atendimento fonoaudiológico neste mesmo hospital. Os critérios de exclusão para o grupo de comparação foram indivíduos com condições de risco para disfagia, como doença neurológica, malformação craniofacial e cirurgia ou radioterapia em região de cabeça e pescoço; risco de disfagia (nota de corte > 3) identificado pelo instrumento de rastreamento de disfagia orofaríngea em idosos (RaDI)^(13)^; e alteração cognitiva ou comportamental que impedisse a realização dos procedimentos do estudo. Considerando que o perfil dos laringectomizados totais da nossa instituição é de indivíduos idosos, optou-se pelo uso do instrumento RaDI por ser apropriado para avaliar o risco de disfagia do grupo de comparação, que por ser pareado ao grupo de estudo, também seria constituído por idosos.

Os critérios de inclusão e exclusão dos grupos foram investigados por meio do prontuário eletrônico, além de entrevista dos participantes quanto às condições de saúde realizada por um fonoaudiólogo.

Os procedimentos do estudo foram realizados por um fonoaudiólogo e consistiram na mensuração da pressão de ponta e de dorso de língua dos participantes, na coleta de dados físicos e antropométricos (sexo, idade, peso, altura e índice de massa corporal) e nível da escala FOIS^(14)^. Além disso, foi aplicado o instrumento SOAL^(8)^ e coletadas informações clínicas (estadiamento da doença, modalidades de tratamento empregadas e tempo de realização da cirurgia) no prontuário dos participantes do grupo de estudo; enquanto, no grupo de comparação, foi aplicado o instrumento RaDI^(15)^.

A mensuração da pressão de língua foi realizada por meio do equipamento de *biofeedback* de pressão de lábios e de língua (PLL Pró-Fono), que permite a extração da média, em kilopascal (kPa), da tarefa de pressão máxima da língua. A mensuração foi realizada em duas porções da língua: primeiramente, na parte anterior; e em seguida, no dorso da língua. Para mensuração da pressão de ponta de língua, o participante foi instruído a segurar o bulbo com uma das mãos, introduzir completamente o bulbo na cavidade oral e posicioná-lo sobre a ponta de língua. Em seguida, o participante foi orientado a pressionar com a ponta de língua o bulbo de ar contra o palato (região alveolar), com o máximo de força possível por três segundos. Para mensuração da pressão de dorso de língua, o participante foi instruído a segurar o bulbo com uma das mãos, introduzir completamente o bulbo na cavidade oral e posicioná-lo sobre o dorso da língua, na porção medial. Em seguida, o participante foi orientado a pressionar com o dorso da língua o bulbo de ar contra o palato duro, com o máximo de força possível por três segundos. Foram realizadas três medidas de pressão de língua, com intervalo de 30 segundos entres as mensurações. Para análise dos dados, foi considerada a média registrada pelo próprio equipamento das três medidas de ponta e de dorso de língua, de acordo com o método empregado por outros pesquisadores^(16)^.

O cálculo amostral foi realizado por meio de estudo piloto com uma parcela da população de interesse considerando um valor de α = 0,05 e um poder de teste (1-β) = 0,80 estimado pelas medidas de pressão de dorso de língua. O número exigido foi de 28 indivíduos, sendo 14 em cada grupo.

A análise dos dados foi realizada por meio do programa SPSS. Os dados categóricos foram apresentados em frequência absoluta e frequência relativa, enquanto os dados numéricos foram apresentados em média e desvio-padrão, em caso de dados com distribuição normal, ou em mediana e intervalos interquartílicos, em caso de dados sem distribuição normal. A normalidade dos dados foi verificada através do histograma e do teste de Shapiro-Wilk.

Para comparação dos dados categóricos entre os grupos, foi utilizado o teste qui-quadrado de Pearson ou o teste exato de Fisher, em caso de célula com frequência menor que cinco. Para comparação dos dados numéricos entre os grupos, foram utilizados o teste t de amostras independentes, em caso de indicação de teste paramétrico, e o teste de Mann-Whitney, em caso de indicação de teste não paramétrico. A indicação do teste paramétrico obedeceu à satisfação da premissa de normalidade de distribuição dos dados em ambos os grupos. Além disso, foi verificada a homogeneidade de variância através do teste de Levene. Para análise de correlação foi utilizado o teste de correlação de Spearman, em virtude da indicação de um teste não paramétrico. O nível de significância estatística adotado foi de 5% (p < 0,05).

## RESULTADOS

Dos 15 indivíduos laringectomizados totais incluídos no estudo, dois foram excluídos, sendo um em virtude da realização de glossectomia e outro em virtude do diagnóstico de demência. A amostra final compreendeu 13 participantes do grupo de estudo e 13 do grupo de comparação. A pontuação total do RaDI no grupo de comparação variou entre 0 e 2 pontos, com mediana de 0 (0 – 1,5) pontos. Todos os participantes do grupo de estudo eram laringectomizados totais e nenhum realizou faringolaringectomia.

As características dos participantes são apresentadas na Tabela 1. Não houve diferença estatisticamente significativa das características dos participantes e do nível da escala FOIS entre os grupos. Em relação ao estadiamento da doença do grupo de estudo, a extensão do tumor variou de T2 a T4; quanto à metástase em linfonodos regionais, houve variação entre N0 e N3; e nenhum participante apresentou metástase à distância.

**Tabela 1:** Características dos participantes dos grupos

Características	Grupo de estudo	Grupo de comparação	Total	p
	(n = 13)	(n = 13)	(n = 26)	
Homens	12 (50%)	12 (50%)	24 (100%)	0,760^a^
Idade (anos)	67,4 ± 6,9	66,7 ± 7,1	67,0 ± 6,9	0,804^b^
Peso (kg)	69,0 ± 12,3	80,4 ± 16,4	74,7 ± 15,3	0,056^b^
Altura (m)	1,72 ± 0,12	1,70 ± 0,08	1,71 ± 0,10	0,617^b^
IMC (Kg/m^2^)	23,9 (20 – 26,2)	26,2 (23,5 – 30,2)	25,6 (21,5 – 27,6)	0,050^c^
Nível da FOIS	7 (6 – 7)	7 (7 – 7)	7 (7 – 7)	0,101^c^
Esvaziamento cervical	13 (100%)	-	-	-
Radioterapia	11 (84,6%)	-	-	-
Quimioterapia	2 (15,4%)	-	-	-
Tempo de cirurgia (meses)	17 (6,5 – 28,5)	-	-	-
Método de voz alaríngea				
- voz esofágica	9 (69,2%)	-	-	-
- laringe eletrônica	2 (15,4%)	-	-	-
- voz traqueoesofágica	2 (15,4%)	-	-	-

Valores apresentados em frequência absoluta (frequência relativa), média ± desvio padrão ou mediana (intervalos interquartílicos)

a Teste exato de Fisher;

b Teste t de amostras independentes;

c Teste de Mann-Whitney

**Legenda:** IMC = índice de massa corporal

A pontuação total do SOAL no grupo de estudo variou entre 0 e 10 pontos, com mediana de 6 (2,5 – 8,5) pontos. A frequência e a intensidade dos sintomas de alteração da deglutição referidos pelos participantes por meio do SOAL são demonstradas na Figura 1. Os sintomas mais frequentes foram relacionados aos itens 9 (*“Você precisa beber líquido para ajudar o alimento descer?”*), presente em 8 participantes, e 1 (*“Na sua opinião, você tem um problema para engolir atualmente?”),* 5 (*“Você tem problema para engolir alimentos duros/secos (pão francês, biscoitos?”*), 7 (*“Após você engolir, sente que o alimento fica parado na garganta?”)* e 11 (*“Você evita certos alimentos porque não consegue engoli-los?”*)*,* presentes em 7 participantes. Quanto à intensidade, os sintomas de alteração da deglutição apontados com maior intensidade foram relacionados aos itens 5 (*“Você tem problema para engolir alimentos duros/secos (pão francês, biscoitos?”*) e 12 (*“Você leva muito tempo para comer uma refeição?”*), apontados por 3 participantes, seguidos pelos itens 9 (*“Você precisa beber líquido para ajudar o alimento descer?”*) e 17 (*“Você se sente constrangido comendo com outras pessoas?”*), apontados por 2 participantes. O item 3 (*“Você tem problema para engolir líquidos engrossados (sopa liquidificada, vitamina)?”*) do SOAL foi o único que não foi pontuado (0 ponto) por nenhum participante.

**Figura 1:** Frequência e intensidade dos sintomas de alteração da deglutição

As medidas da pressão de dorso e de ponta de língua entre os grupos são apresentadas na Tabela 2. Houve diferença estatisticamente significativa da pressão de dorso de língua entre os grupos, apesar de não haver diferença da pressão de ponta de língua entre os grupos.

**Tabela 2:** Comparação da pressão de língua entre os grupos

Pressão (kPa)	Grupo de estudo	Grupo de comparação	Total	p
Dorso de língua	41,2 ± 18,7	27,9 ± 9,3	34,6 ± 16,0	**0,034** a
Ponta de língua	33,7 (23,8 – 49,3)	29,1 (22,5 – 35,7)	30,0 (23,2 – 40,8)	0,287^b^

Valores apresentados em média ± desvio padrão ou mediana (intervalos interquartílicos).

a Teste t de amostras independentes;

b Teste de Mann-Whitney

A avaliação da correlação entre a pressão de língua e a autoavaliação da deglutição do grupo de estudo é apresentada na Tabela 3. Não houve correlação estatisticamente significativa entre as medidas de pressão da língua e a autoavaliação da deglutição, apesar de haver correlação positiva de força moderada entre a pressão de ponta e a pressão do dorso da língua.

**Tabela 3:** Correlação entre pressão de língua e autoavaliação da deglutição

Pressão de língua	Pressão de dorso	Pontuação total do SOAL
Pressão de ponta	r = 0,665; **p = 0,013**	r = - 0,168; p= 0,583
Pressão de dorso	-	r = - 0,303; p = 0,314

Teste de correlação de Spearman

## DISCUSSÃO

Este estudo mensurou e comparou as medidas de pressão de ponta e de dorso de língua em indivíduos com e sem LT. Além disso, o estudo descreveu a autoavaliação da deglutição de indivíduos submetidos à LT e analisou se existe correlação entre as medidas de pressão de língua e da autoavaliação da deglutição destes indivíduos.

Em relação à medida de pressão de língua, nossos resultados revelaram que indivíduos submetidos à LT exibem maior pressão de dorso de língua comparado aos indivíduos sem LT. Por outro lado, assim como outros pesquisadores, este estudo não evidenciou diferença estatisticamente significativa da medida de pressão de ponta de língua entre indivíduos com e sem LT^(17)^.

Outras pesquisas mensuraram a pressão máxima da ponta de língua de indivíduos laringectomizados totais, correspondentes a 44,1±11,3 kPa^(9)^ e 50,6 (IC95% 45,1-56,1) kPa^(2)^, medidas superiores a encontrada neste estudo. Entretanto, essas pesquisas utilizaram o instrumento IOPI, que determina a pressão máxima (pico de pressão) alcançada, diferentemente do instrumento PLL, utilizado neste estudo, que determina a média da pressão de língua durante a tarefa de pressão máxima.

A língua é uma estrutura fundamental para a deglutição, já que além do seu importante papel na fase oral, a ação desta estrutura é determinante para o desempenho da fase faríngea. A aproximação da base de língua à parede posterior de faringe é crucial para a geração da pressão faríngea e direcionamento eficiente do bolo^(18)^. Entretanto, indivíduos submetidos à LT podem apresentar prejuízo do movimento de posteriorização da base de língua^(12)^. Sendo assim, é possível que a maior pressão de dorso de língua, evidenciada neste estudo, nos laringectomizados totais seja um ajuste compensatório desenvolvido em decorrência das alterações anatomofisiológicas da função de deglutição acarretadas pela cirurgia.

Além disso, pode-se cogitar a importância da pressão de dorso de língua para a realização das técnicas de introdução de ar no esôfago para a aquisição da voz esofágica, método mais comumente empregado na população deste estudo. Dessa forma, é possível que o aumento da pressão de dorso de língua nestes indivíduos também seja um ajuste desenvolvido para o aprendizado da voz esofágica. Apesar de um estudo não ter encontrado diferença na força de língua entre laringectomizados totais proficientes na voz esofágica e indivíduos sem LT^(17)^, foi observado que laringectomizados totais usuários de laringe eletrônica podem apresentar menor força de língua comparados aos indivíduos sem LT^(19)^.

Nossos resultados revelaram uma correlação positiva de força moderada entre a pressão de ponta de língua e a pressão de dorso de língua nos indivíduos laringectomizados totais, ou seja, quanto maior a pressão de ponta de língua, maior é a pressão de dorso de língua. Este resultado é facilmente justificado pelo fato de que tais porções anatômicas são estruturalmente dependentes, já que são porções da mesma estrutura anatômica. Portanto, espera-se que indivíduos que apresentem maior pressão de uma porção da língua, também apresentem maior pressão da outra porção desta mesma estrutura.

O escore do SOAL varia de 0 a 34 pontos e pontuações mais altas indicam maiores problemas autorreferidos relacionados à deglutição^(4)^. Um estudo encontrou escore médio de 8,6 em indivíduos com dieta normal e 18,3 em indivíduos com dieta modificada ou sem dieta por via oral^(4)^. De acordo com Govender et al.^(20)^, um indivíduo sem características adversas no exame de videofluoroscopia teria um escore previsto de aproximadamente 5 pontos no SOAL. A mediana do SOAL encontrada nos participantes do nosso estudo foi ligeiramente maior (6 pontos), o que indica que parte considerável destes indivíduos apresenta sintomas relacionados à dificuldade de deglutição, porém é possível que tais indivíduos não apresentem características adversas no exame de videofluoroscopia. Além disso, os nossos resultados revelaram um escore do SOAL menor do que o encontrado por outros estudos, com média de 11,3±7,6^(4)^ e 13,6 (IC95% 10,8-16,3) pontos^(2)^, o que indica um pior resultado da autoavaliação da deglutição nestes outros estudos. Uma possível razão que pode justificar esta diferença está relacionada ao tempo de realização do tratamento da doença, já que os efeitos tardios da radioterapia, como fibrose e estenose, podem impactar negativamente a função de deglutição. Enquanto os nossos participantes exibiram uma mediana de 17 meses de realização da cirurgia, os outros estudos exibiram mediana de 39^(4)^ e 47 meses^(2)^. Além disso, outra possível justificativa para esta diferença relaciona-se ao fato dos participantes do nosso estudo realizarem fonoterapia, o que pode contribuir para a redução dos sintomas de alteração da deglutição.

Em consonância com a literatura, a maioria dos participantes deste estudo referiu sintomas relacionados à dificuldade de deglutição, associados principalmente à necessidade de beber líquidos para ajudar o alimento a descer^(3)^. Dificuldade de deglutição de alimentos duros ou secos, alimento parado na garganta e evitar certos alimentos porque não consegue degluti-los também foram sintomas muito frequentes em nossa população. Tais sintomas corroboram os achados de outros pesquisadores^(2-4)^ e já eram esperados, uma vez que as mudanças anatômicas e fisiológicas após a cirurgia de LT predispõem estes indivíduos a apresentarem resíduo na neofaringe^(21)^. De acordo com a literatura, o aumento do tamanho e da viscosidade do bolo implica no aumento do resíduo faríngeo nos laringectomizados totais^(22)^. Por outro lado, sintomas relacionados à dificuldade de deglutição de líquidos, líquidos engrossados e alimentos pastosos ou macios foram ausentes ou menos frequentes em nossa população, em concordância com outros estudos^(2,4)^.

Apesar da nossa hipótese de que indivíduos com menor pressão de língua pudessem apresentar pior pontuação da autoavaliação da deglutição, este estudo não encontrou correlação entre a medida da pressão de língua e a pontuação da autoavaliação da deglutição de indivíduos laringectomizados totais. Corroborando com estes dados, outros pesquisadores não encontraram diferença do pico de pressão de base de língua entre laringectomizados totais com e sem sintomas de disfagia^(22)^.

Este estudo apresenta limitações, em virtude do tamanho reduzido da amostra. Destaca-se, portanto, a possibilidade de erro tipo II na análise de comparação da pressão de ponta de língua entre indivíduos com e sem LT e da correlação entre a pressão de língua e a autoavaliação da deglutição dos indivíduos laringectomizados totais. Além disso, este estudo não considera a técnica de fechamento cirúrgico empregada nos participantes, que pode interferir na biomecânica da deglutição^(22)^ e, consequentemente, nos aspectos avaliados neste estudo.

## CONCLUSÃO

As medidas de pressão de dorso e de ponta de língua dos indivíduos laringectomizados totais participantes deste estudo alcançam, em média, 41 kPa e 34 kPa respectivamente. Pode-se concluir que estes indivíduos apresentam maior pressão de dorso de língua comparado aos indivíduos sem LT, apesar de não haver diferença na pressão de ponta de língua entre os indivíduos com e sem LT participantes deste estudo. Além disso, este estudo não evidenciou correlação entre a pressão de língua e a autoavaliação da deglutição de indivíduos laringectomizados totais, embora haja correlação entre a pressão de ponta de língua e a pressão de dorso de língua nesta população. A maioria destes indivíduos apresenta sintomas de transtornos de deglutição relacionados à necessidade de ingestão de líquidos para auxiliar o transporte do bolo, à dificuldade de deglutição de alimentos duros ou secos, à sensação de alimento parado na garganta e à necessidade de evitar alguns alimentos em virtude da dificuldade de deglutição.
